# An inactivated vaccine made from a U.S. field isolate of porcine epidemic disease virus is immunogenic in pigs as demonstrated by a dose-titration

**DOI:** 10.1186/s12917-015-0357-1

**Published:** 2015-03-15

**Authors:** Emily A Collin, Srivishnupriya Anbalagan, Faten Okda, Ron Batman, Eric Nelson, Ben M Hause

**Affiliations:** Newport Laboratories Inc., Worthington, MN USA; Department of Veterinary and Biomedical Sciences, South Dakota State University, Brookings, SD USA; Present Address: Veterinary Diagnostic Laboratory, Kansas State University, Manhattan, KS USA; National Research Center, Giza, Egypt

**Keywords:** Porcine, Epidemic, Diarrhea, Virus, PEDV, Inactivated, Vaccine, Immunogenicity

## Abstract

**Background:**

Porcine epidemic diarrhea virus (PEDV), a highly pathogenic and transmissible virus in swine, was first detected in the U.S. in May, 2013, and has caused tremendous losses to the swine industry. Due to the difficulty in isolating and growing this virus in cell culture, few vaccine studies using cell culture propagated PEDV have been performed on U.S. strains in pigs. Therefore, the objective of this study was to evaluate the humoral immune response to the selected inactivated PEDV vaccine candidate in a dose-titration manner.

**Results:**

PEDV was isolated from a pig with diarrhea and complete genome sequencing found >99% nucleotide identity to other U.S. PEDV. Inactivated adjuvanted monovalent vaccines were administered intramuscularly to five week old pigs in a dose titration experimental design, ranging from 6.0-8.0 log_10_ tissue culture infective dose (TCID_50_/mL), to evaluate immunogenicity using a fluorescent foci neutralization assay (FFN), fluorescent microsphere immunoassay (FMIA), and enzyme-linked immunosorbent assay (ELISA) on sera. Pigs vaccinated with 8.0 log_10_ TCID_50_/mL inactivated virus showed significantly higher FFN titers as well as FMIA and ELISA values than 6.0 log_10_ TCID_50_/mL vaccinates and the negative controls.

**Conclusions:**

These results demonstrate the immunogenicity of a PEDV inactivated viral vaccine with a U.S. strain via dose-titration. A future vaccination-challenge study would illustrate the efficacy of an inactivated vaccine and help evaluate protective FFN titers and ELISA and FMIA responses.

## Background

Porcine epidemic diarrhea virus (PEDV) circulated throughout Europe and Asia during the past three decades before being detected in swine in the United States in May, 2013 [1-7]. Since its introduction to the U.S., PEDV has been identified in 33 states by the National Animal Health Laboratory Network, as of December, 2014 (www.aasv.org). It is characterized by watery diarrhea, vomiting, dehydration, and high mortality rates in suckling pigs [8-10]. The U.S. PEDV strains are phylogenetically subgroup IIa, which is similar to PEDV circulating in Asia in 2011 and 2012 [6,7].

Modified-live vaccines (MLVs) have long been used in Asia for the control of PEDV [11-13]. The strain 83P-5, attenuated by one-hundred cell culture passages, is a subgroup I isolate that has been licensed in Japan as an attenuated live PEDV vaccine [13]. During the attenuation process, this strain acquired fourteen amino acid changes in the immunodominant spike (S) protein, which is critical for virus binding to cell receptors and is the target of neutralizing antibodies [14-19]. The live attenuated DR13 vaccine strain of PEDV, whose parent strain was a subgroup II had thirteen of these fourteen mutations as well, and subsequently clustered with subgroup I [13]. Serial passage of 83P-5 in Vero cells resulted in attenuation of virulence *in vivo* and the strong selection for the viral spike (S) gene was associated with these phenotypic changes.

Classically attenuated cell culture passaged PEDV also shows mutations in open reading frame 3 (ORF) and changes to restriction fragment length polymorphism (RFLP) cut patterns, which have been used to distinguish MLV from field strains [10,20]. *In vivo*, high-passage (x > 100) MLVs were attenuated in sows and piglets while still capable of inducing a robust immune response [20]. While attenuated in their ability to cause disease, the safety of using MLV has been questioned, as MLV are shed in the environment. Virus was detected in feces of 3-day old piglets up to seven days after oral inoculation with DR13 passage 100 [12,21] . In 2010, PEDV was isolated from diarrheic pigs in China that had a close phylogenetic relationship to two MLV vaccines, suggesting it may have evolved from a MLV [22].

While modified live vaccines generally elicit a more robust and protective immune response than inactivated virus vaccines [13], long-term efficacy is often lacking due to viral mutations and accompanying antigenic changes [23]. In late 2010, China experienced a severe outbreak of PEDV in suckling pigs, causing drastic economic losses [24]. This outbreak was caused by a strain with a phylogenetically distinct S gene from other Chinese strains and from vaccine strain CV777 [24]. In 2012, the PEDV infection rates in vaccinated herds in China increased dramatically. Phylogenetic analysis of new variants from the outbreak showed insertions and deletions in antigenic regions of the S gene that may have influenced the efficacy of the CV777 MLV [25]. Investigation into whether an inactivated vaccine can elicit a protective immune response could lead to the development of vaccines more closely related to field strains and avoid potential antigenic changes due to excessive *in vitro* cultivation.

There are currently two conditionally licensed PEDV vaccines in the U.S, with label claims for use in sows; an inactivated virus vaccine and an alphavirus vectored subunit vaccine. With mortality rates as high as 100% in suckling piglets and total losses estimated over 5 million animals in the U.S. in less than one year, PEDV vaccines are critically needed (www.aasv.org). The U.S. Department of Agriculture (USDA) allows for the production of autogenous vaccines to address emerging diseases; however the difficulty in isolating PEDV in cell culture increases the difficulty in producing efficacious inactivated vaccines. Here, PEDV was isolated from pooled intestinal homogenate and passaged in cell culture. Inactivated cell culture derived viral vaccines were immunogenic when administered to naïve pigs. To our knowledge, this is the first demonstration of immunogenicity of an inactivated U.S. PEDV vaccine trial in pigs in the U.S.

## Methods

### Ethics statement

Swine studies were performed at Newport Laboratories and were approved by the Newport Laboratories’ Institutional Animal Care and Use Committee.

### Virus isolation

In May, 2013, intestines from pigs in Iowa experiencing PEDV-like symptoms were submitted to Newport Laboratories for diagnostic testing. Intestines were homogenized in phosphate buffered saline and debris was removed by centrifugation at 10,000 × g for 10 minutes followed by filtration through a 0.2 μm filter. Virus isolation was performed on Vero (ATCC® CCL-81™), Vero 76 (ATCC® CRL-1586™), and MARC-145 (M145) cells [26]. All cells were maintained in Dulbecco’s modification of Eagles medium (DMEM) with five percent fetal bovine serum and one percent L-glutamine. Confluent monolayers were washed three times with DMEM without serum prior to inoculation. For the initial infection of cells in 12-well plates, 200 μL of inoculum was adsorbed at 37°C with + 5% CO_2_ for 1–2 hours with small amount of viral growth media (DMEM with 0.75 μg/mL ), L-1-Tosylamide-2-phenylethyl chloromethyl ketone (TPCK)-treated trypsin, and Normocin™ antibiotic (Invivogen)). The inoculum was rinsed from the plates with viral growth media and the cells were refed with viral growth media. Plates were incubated up to 5 days before being frozen, thawed, and passaged. Subsequent passages were performed by inoculating 200 μL of cell culture harvest onto confluent monolayers in 12-well plates. Viral replication was verified by Real time reverse transcription polymerase chain reaction (rt-RT-PCR) (below) and indirect immunofluorescence (IFA). Viral cultures were scaled up in M145 25 cm^2^ flasks and 1700 cm^2^ roller bottles, resulting in NPL PEDV 2013 P10.1PEDV.

### Indirect immunofluorescence

IFA was performed on Vero or M145 96-well monolayers. Infected wells were fixed in cold ethyl alcohol and polyclonal rabbit anti-PEDV nucleoprotein (NP) antiserum (South Dakota State University Animal Disease Research and Diagnostic Laboratory (SDSU)) was added at 1:500. Cells were rinsed and then incubated with fluorescein isothiocyanate (FITC) labeled goat anti-rabbit IgG (Jackson Immunoresearch) at a dilution of 1:50, and then read using a fluorescent microscope. Tissue culture infective dose (TCID_50_/mL) was calculated using the Spearman-Karber method.

### Molecular analysis

Viral RNA from cell culture passages was extracted by using the MagMAX™-96 viral RNA isolation kit (Life Technologies) according to the manufacturer’s instructions. rt-RT-PCR was performed by using QIAGEN Quantitect® RT-PCR with the following PEDV primers and probe: PEDV forward: 5’-ACG TCC GTA ACA CCT TCA AG -3’ , PEDV reverse: 5’-GCT AGT GCC TGT ACC ATA GAT C-3’ , and PEDV Probe: 5'-/5HEX/ CGT GCC AGT AAT CAA CTC ACC CTT TGT /3IABkFQ/-3'. For analytical purposes, negative samples were assigned a Ct value of 37.1, which corresponds to the detection limit of the method (approximately −1.0 TCID_50_/mL). Method specificity was assessed by using various porcine enteric viruses, including transmissible gastroenteritis virus, group A rotavirus and porcine enterovirus, and no cross-reaction was observed. A standard curve was generated by serial dilution of M145 cell harvests containing 5.7 log_10_ TCID_50_/mL of PEDV, as determined by titration on M145 cells.

### RNA isolation for next generation sequencing

M145 cells that showed 100% cytopathic effects (CPE) following virus infection at passage x + 9 were used for RNA extraction for sequencing. 20 mL of cell culture supernatant was filtered using the 0.2 μm bottle top filters (Thermo Scientific, Lenexa, Kansas). The filtrate was centrifuged at 50,000 × g for 2 hours. Supernatant was discarded and the pellet was suspended in 1000 μL of water. Samples were concentrated to a final 100 μL volume using Amicon® ultra centrifugal filters (0.5 mL; 50KDa) (Millipore, Tullagreen, Ireland). Cellular DNA and RNA were removed by incubation with DNase I (25 units) (New England Biolabs, NEB, Ipswich, MA) and RNase A (25units) (Qiagen, Valencia, CA) at 37°C for 1 hour. RNA was extracted using Trizol® LS Reagent (Life Technologies, Grand Island, NY) according to manufacturer’s instructions. The pellet containing RNA was resuspended in 20 μL of sterile H_2_O.

### Sequencing and data analysis

10 μg of total RNA was depleted of ribosomal RNA using GeneRead™ rRNA depletion kit (Qiagen) and RNA sequencing libraries were generated using the Ion Total RNA-seq kit v2 (Ion Torrent™, Life Technologies) according to manufacturer’s instructions. Sequencing was carried out using Ion Personal Genome Machine® (PGM) sequencing platform (Life Technologies, Grand Island, NY) as previously described [27]. Sequence reads were assembled into contigs using the SeqMan® NGen program (DNAstar, Madison, WI). Phylogenetic analysis on full genome sequences was performed using MEGA™ 6.0 software using Maximum Likelihood analysis with 1000 bootstrap replicates to verify tree topology. Sequence alignments were performed using the ClustalW algorithm in MegAlign (DNAstar, Madison WI). The complete genome of NPL PEDV 2013 P10.1 was compared to the sequence derived from the original clinical sample [Genbank:KJ778615] and various reference strains. The reference strains included: CV777 [Genbank:EF353511] from Belgium; DR13 attenuated [Genbank:JQ023162], DR13 virulent [Genbank:JQ023161], and SM98 [Genbank:GU937797] from South Korea; LZC [Genbank:EF185992], JS2008 [Genbank:KC109141], and CHS [Genbank:JN547228] from China; CO13 [Genbank:KF272920], MN [Genbank:KF468752], and a variant strain OH851 [Genbank:KJ399978] from the United States. The genome sequence for NPL PEDV 2013 P10.1 was deposited in GenBank under the accession number KM052365 [Genbank:KM052365].

### Assessment of immunogenicity in swine

Swine vaccination studies were approved by the Institutional Biosafety Committee, under Institutional Animal Care and Use Committee (IACUC) guidelines. The studies were performed at Newport Laboratories under biosafety level 1. Sixty pigs approximately 4 weeks of age were obtained from a commercial high-health herd. They were of mixed sexes of crossbred American Yorkshire-Landrace-Duroc. Prior to study commencement pigs were verified as serologically negative to PEDV by FFN and IFA. Pigs were also negative for PEDV shedding by rt-RT-PCR on fecal swabs. Pigs were divided into 8 vaccination groups of 5–9 pigs and a non-vaccinated control group of 5 pigs, co-mingled among two different rooms. Pigs were allowed 1 week to acclimate prior to study commencement.

Virus NPL PEDV 2013 P10.1 (6.6 log_10_ TCID_50_/mL) was inactivated and concentrated 30X for use as vaccine. Inactivation was performed by the addition of 0.1 M binary ethyleneimine (BEI) to a final volume of 5% and incubating at 37°C for 24 hours. Excess BEI was neutralized with sodium thiosulfate. Virus inactivation was verified by passaging the inactivated fluids three times on permissive cells, resulting in no evidence of CPE or increase in rt-RT-PCR titer. Concentration was performed using a 10kD hollow fiber filter (Spectrum Labs). Due to the space constraints and the number of study groups, vaccination groups receiving 8.0 log_10_ TCID_50_/mL antigen consisted of more pigs than groups receiving lower levels of antigen as they were anticipated to show the most robust immune response (Table 1). Groups 1–3 were vaccinated intramuscularly (IM) in the neck with 2 mL of 8.0, 7.0 or 6.0 log_10_ TCID_50_/mL, respectively, of inactivated virus. Groups 5–7 were vaccinated IM in the neck with 2 mL of 8.0, 7.0 or 6.0 log_10_ TCID_50_/mL, respectively, of inactivated virus treated with Triton® X-100 (added to 0.1% and incubated at room temperature 30 minutes) (Sigma). Groups 4 and 8 were vaccinated in the rear flank with 8.0 log_10_ TCID_50_/mL of inactivated virus and inactivated virus treated with Triton® X-100, respectively. This was done to evaluate any potential changes in immune response due to a change in vaccination site. All vaccines were formulated to contain 67% TS6, a proprietary oil in water adjuvant. Pigs were vaccinated on days 0 and 21 and observed for adverse vaccine reactions for one hour. Animals were also observed daily for signs of disease. Serum was collected on days 0, 21 and 35, and the study was terminated at day 35. Fecal swabs were collected three days post vaccination and were tested by rt-RT-PCR to confirm absence of PEDV shedding.

**Table 1:** **Vaccination groups and treatment**

**Group**	Vaccine*	**Pigs**
0	Negative control	5
1	8.0 IM	8
2	7.0 IM	5
3	6.0 IM	5
4	8.0 RF	9
5	8.0 IM + Triton® X-100	9
6	7.0 IM + Triton® X-100	5
7	6.0 IM + Triton® X-100	5
8	8.0 RF + Triton® X-100	9

*PEDV titer in vaccine prior to inactivation (log_10_ TCID_50_/mL) and route of administration (IM, intramuscular neck; RF, rear flank).

### Serology

The fluorescent foci neutralization assay (FFN) was performed at SDSU using a National Veterinary Services Laboratory (NVSL) reference isolate, USA/Colorado/2013 (CO/13). Briefly, test and control serum samples were heat inactivated at 56°C for 30 minutes, then serially diluted in serum-free Modified Eagles Medium (MEM) containing 1.0 μg/mL TPCK treated trypsin in 96-well plates with a final volume of 100 μL/well. Next, 100 μL of PEDV stock diluted to 100–200 fluorescent focus units (FFU)/100 μL was added to each well and plates were incubated at 37°C for 1 hour. Plates containing confluent 3 day old monolayers of Vero-76 cells were washed 3 times with serum-free MEM prior to transfer of the serum/virus mixtures to corresponding wells of these plates. After 1 hour incubation at 37°C, the serum/virus mixture was removed, monolayers washed once with serum-free MEM and 150 μL/well replacement media (MEM with 1.0 μg/mL TPCK treated trypsin) was added to each well. Plates were incubated 24 hours at 37°C, then monolayers fixed for 15 minutes with 80% acetone in water, dried and stained with fluorescein conjugated PEDV anti-nucleoprotein (NP) monoclonal antibody SD6-29. Titers were reported as the reciprocal of the greatest serum dilution resulting in a 90% or greater reduction in FFU relative to virus control well. A FFN titer <20 was considered negative.

Enzyme-linked immunosorbent assay (ELISA) was performed at the University of Minnesota (UMN) Veterinary Diagnostic Laboratory. The assay utilizes a recombinant PEDV NP antigen and samples with a sample to positive ratio (S/P) value greater than 0.5 are considered positive. An experimental ELISA using a recombinant PEDV NP was also performed at SDSU to verify the UMN results. The SDSU cutoff for positive results was an S/P of 0.4, according to ROC analysis using Medcalc software (unpublished conference proceedings). Both ELISAs detected only swine IgG.

To further investigate the immune response to the inactivated PEDV vaccine, a fluorescent microsphere immunoassay (FMIA) was performed at SDSU using the same purified, full-length 51 kDa PEDV nucleoprotein antigen used in the ELISA. Briefly, the full length nucleocapsid open reading frame of PEDV was cloned into the pET-28a prokaryotic expression vector (Novagen). Recombinant proteins were expressed as His-tagged fusion proteins and purified using Ni-NTA agarose column chromatography prior to fluorescent microsphere coupling. The FMIA was performed as previously described [28,29]. Coupled microspheres were analyzed through a dual-laser Bio-Rad Bio-Plex 200 instrument. The mean fluorescence intensity (MFI) for 100 microspheres corresponding to each individual bead analyte was recorded for each well. All reported MFI measurements were normalized via F - F_0_, where F_0_ was the background signal determined from the fluorescence measurement of a test sample in uncoated beads and *F* was the MFI for a serological test sample in antigen-coated beads.

### Statistical analysis

Statistical analysis was performed using SPSS 14 software. One-way ANOVA and Tukey HSD was performed on all groups, using harmonic mean sample size of 6.171 to account for unequal group sizes. Also, a comparison between the groups that had the same titer of the virus and different treatment was done by t-test.

## Results

### Virus isolation

The rt-RT-PCR Ct value of the PEDV positive intestinal homogenate was 21.4. After initial isolation attempts on Vero and Vero 76 cell lines, passaging of samples continued on Vero cells as viral replication was evident by rt-RT-PCR. CPE was evident after two passages and confirmed as PEDV by rt-RT-PCR and IFA. The Ct values for passages x + 1 through x + 5 ranged from 17.8-23.5. Cultures were scaled to a T25 Vero flask for x + 6 (17.97 Ct, 4.4 log_10_ TCID_50_/mL). Cell cultures were adapted to M145 cells at x + 7 (18.55 Ct, 4.4 log_10_ TCID_50_/mL) and x + 8 (23.31 Ct and 5.2 TCID_50_/mL) due to their USDA-licensed status for autogenous vaccine production. After two passages in M145 25 cm^2^ flasks, the culture was scaled up to 1700 cm^2^ roller bottles of M145 cells. This passage, X + 9, had a Ct = 21.2 and a titer of 6.6 log_10_ TCID_50_/mL as determined by IFA. The isolated PEDV was designated NPL PEDV 2013 P10.1.

### Genetic analysis

**Figure 1:**
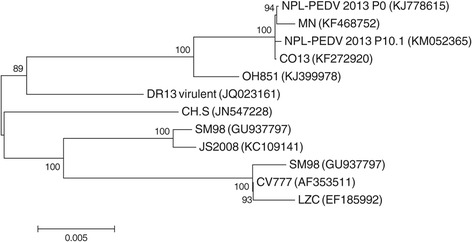
**Phylogenetic analysis of 12 full-length porcine epidemic diarrhea virus genomes.** Phylogenetic analysis was performed using MEGA 6.0 software using Maximum Likelihood analysis with 1000 bootstrap replicates to verify tree topology. Genbank accession numbers are shown in parentheses. Bootstrap values are shown above and to the left of major nodes.

Phylogenetic analysis of complete genome sequences showed >99% identity to U.S. PEDV virus CO/13 [Genbank:KF272920] and the original intestinal sample [Genbank:KJ778615]. The Minnesota isolate [Genbank:KF468752] and an isolate from Ohio [Genbank:KJ408801] were also closely related to the NPL PEDV2013 strain (Figure 1). The ORF1ab, S, ORF3, envelope (E), membrane (M), and NP genes of eleven PED reference viruses were aligned and the percent nucleotide identity to NPL PEDV2013 P10.1 was determined (Table 2). ORF3 showed the greatest divergence, with 93.1-100% nucleotide identity. The S gene was the next most divergent, with 93.5-99.9% nucleotide identity. Amongst the US strains, ORF3, E, M, and NP were highly conserved with greater than 99.8% nucleotide identity. The S gene showed the greatest variability amongst U.S. strains, with OH851 having 96.9% identity to NPL PEDV 2013 P10.1 [30].

**Table 2:** **Genogroup and percent nucleotide identity of reference porcine epidemic diarrhea viruses to NPL PEDV 2013 P10.1**

**Virus (accession number)**	**Genogroup**	**ORF 1ab**	**S**	**ORF3**	**E**	**M**	**NP**
CHS (JN47228)	G1	98.0	93.8	98.2	96.5	98.1	96.8
CO13 (KF272920)	G2	100.0	99.9	99.9	100.0	100.0	100.0
CV777 (AF353511)	G1	97.3	94.0	96.9	97.0	98.2	96.0
DR13 Attenuated (DQ462404)	G1	97.8	93.6	93.1	96.7	97.9	96.8
DR13 Virulent (JQ023161)	G2	98.2	95.0	98.5	98.3	98.4	97.4
JS2008 (KC109141)	G1	98.0	94.2	93.1	96.1	97.8	96.8
LZC (EF185992)	G1	97.2	93.5	95.6	96.1	97.2	95.8
MN (KJ468752)	G2	99.8	99.7	100.0	100.0	100.0	100.0
OH851 (KJ399978)	G2	99.5	96.9	100.0	100.0	99.9	99.8
SM98 (GU937797)	G1	97.2	93.7	96.8	96.1	98.1	95.9
NPL PEDV2013 p0 (KJ778615)	G2	100.0	99.8	100.0	100.0	100.0	100.0

### Pig vaccination and serology

All pigs in the study were confirmed seronegative for PEDV antibodies at day 0 by IFA and FFN (data not shown). Pig fecal swabs collected on day 3 post vaccination were negative for PEDV, further confirming that no infectious PEDV was present in the vaccine. No adverse reactions were noted following vaccination on days 0 and 21.

All vaccine groups had positive mean titers by the FFN (Table 3). Post Hoc analysis of the FFN results showed that all 8.0 log_10_ TCID_50_/mL groups [1,4,5,8], along with 7.0 log_10_ TCID_50_/mL Triton® X-100 treated group (group 6), had statistically significant higher mean FFN titers than the control group 0 at p = 0.05 level and did not have a significant difference in means compared to each other per Tukey HSD. No groups were statistically similar to the control group by Tukey HSD analysis (using harmonic mean sample size of 6.171 due to unequal group sizes). The t-test was also performed on groups that had the same titer of virus and different treatment (Table 4). There was no statistical difference in FFN titers between vaccination groups of the same titer with and without Triton® X-100 treatment for the FFN assay.

**Table 3:** **One way Anova and Tukey HSD**

**Group**		**FFN** ^**ǂ**^	**UM ELISA** ^**#**^	**SDSU ELISA** ^**#§**^	**FMIA** ^**£**^
**0 (control)**	**Mean**	1.93 ^D^	0.00^B^	0.05^c^	0.04^C^
**N = 5**	**Std.Deviation**	2.66	0.00	0.05	0.04
	**Std. Error**	1.19	0.00	0.02	0.02
**1 - 8.0 log**_**10**_ **TCID**_**50**_ **/mL**	**Mean**	7.32^*A,B^	1.06^*A^	1.79^*A^	1.14^*A^
**N = 8**	**Std.Deviation**	1.2	0.17	0.79	0.04
	**Std. Error**	0.42	0.06	0.28	0.01
**2 - 7.0 log**_**10**_ **TCID**_**50**_ **/mL**	**Mean**	5.06^B,C^	0.29^B^	0.57^B^	0.51^*B^
**N = 5**	**Std.Deviation**	3.24	0.39	0.54	0.42
	**Std. Error**	1.45	0.17	0.24	0.19
**3 - 6.0 log**_**10**_ **TCID**_**50**_ **/mL**	**Mean**	4.66^C^	0.01^B^	0.07^C^	0.08^C^
**N = 5**	**Std.Deviation**	2.74	0.02	0.04	0.07
	**Std. Error**	1.23	0.01	0.02	0.03
**4 - 8.0 log**_**10**_ **TCID**_**50**_ **/mL (Flank)**	**Mean**	7.99^*A^	1.04^*A^	2.11^*A^	1.16^*A^
**N = 9**	**Std.Deviation**	1.32	0.40	0.26	0.03
	**Std. Error**	0.44	0.13	0.09	0.01
**5 - 8.0 log**_**10**_ **TCID**_**50**_ **/mL (Triton® X-100)**	**Mean**	6.99^*A,B^	0.00^B^	0.04^C^	0.06^C^
**N = 9**	**Std.Deviation**	1.80	0.01	0.03	0.07
	**Std. Error**	0.60	0.00	0.01	0.02
**6 - 7.0 log**_**10**_ **TCID**_**50**_ **/mL (Triton® X-100)**	**Mean**	6.52^*A,B,C^	0.11^B^	0.03^C^	0.04^C^
**N = 5**	**Std.Deviation**	0.84	0.03	0.04	0.05
	**Std. Error**	0.37	0.011	0.02	0.23
**7 - 6.0 log**_**10**_ **TCID**_**50**_ **/mL (Triton® X-100)**	**Mean**	5.12^B,C^	0.01^B^	0.36^C,B^	0.14^C^
**N = 5**	**Std.Deviation**	0.84	0.02	0.59	0.13
	**Std. Error**	0.37	0.01	0.27	0.06
**8 - 8.0 log**_**10**_ **TCID**_**50**_ **/mL (Triton® X-100, Flank)**	**Mean**	7.54^*A^	0.14^B^	0.06^C^	0.02^C^
**N = 9**	**Std.Deviation**	1.20	0.41	0.45	0.03
	**Std. Error**	0.40	0.14	0.02	0.11

^A,B,C,D^ Tukey HSD lists different letters between groups whose means that are statistically significant. Those with same letters means no significant difference among their means. Tukey HSD used harmonic mean sample size = 6.171 to account for differences in group sizes.

^ǂ^The FFN results were log2 transformed before analysis.

^#^The ELISA results are sample to positive (S/P) ratios. The UMN ELISA cutoff is 0.5. ^#§^The SDSU cutoff for ELISA is S/P of 0.4.

^£^The FMIA MFI cutoff is 0.1.

*The mean difference is significant at the 0.05 level compared to the control group.

**Table 4:** **T-test comparison between groups with same titer and different treatment (with and without Triton® X-100 treatment), results considered significant at p <0.05 level**

**Group**	**FFN**	**UM ELISA**	**SDSU ELISA**	**FMIA**
2,6	.356	.143	.057	.038
3,7	.727	.986	.305	.438
1,5	.657	.000	.000	.000
4,8	.466	.000	.000	.000

Only one of the 9 animals in group 8, the 8.0 log_10_ TCID_50_/mL of inactivated Triton® X-100 treated virus to the rear flank, showed positive ELISA results at UMN (data not shown). To further clarify these results, an ELISA test was performed at SDSU and the results supported the UMN results in that no anti-nucleoprotein antibody from the Triton® X-100 treated groups was recognized. The ELISA results showed highly significant increases in S/P ratios in the 8.0 log_10_ TCID_50_/mL of inactivated virus to the IM neck and rear flank (groups 1 and 4, respectively) compared to control group 0 for both UMN and SDSU assays (Table 3). There was no significance between groups 1 and 4 by Tukey HSD, but both had significantly higher results compared to the remaining groups for the UMN ELISA. For the SDSU ELISA, group 2 (7.0 log_10_ TCID_50_/mL) also had significantly higher results than groups 3, 5, 6, and 8, per Tukey HSD. The t-test shows significant differences in ELISA results for the 8.0 log_10_ TCID_50_/mL groups 1 and 5 (IM, with and without Triton® X-100, respectively), as well as the 8.0 log_10_ TCID_50_/mL groups 4 and 8 (flank, with and without Triton® X-100, respectively). Also, the SDSU ELISA showed near significant difference between the 7.0 log_10_ TCID_50_/mL groups 2 and 6 (with and without Triton® X-100, respectively) with a p value of 0.057.

FMIA was performed and groups 1, 2, and 4 (8.0 log_10_ TCID_50_/mL and 7.0 log_10_ TCID_50_/mL IM and 8.0 log_10_ TCID_50_/mL flank) and MFI values were significantly higher than the control by Tukey HSD analysis (Table 3). Again, the 8.0 log_10_ TCID_50_/mL vaccine groups and the 7.0 log_10_ TCID_50_/mL group without Triton® X-100 had statistically higher MFI than the Triton® X-100 treated groups by t-test (Table 4). Group 2, the 7.0 log_10_ TCID_50_/mL group, had statistically lower MFI than groups 1 and 4, and significantly higher MFIs than all other groups. The 6.0 log_10_ TCID_50_/mL groups [3,7] MFI showed no difference in Triton® X-100 treatment by t-test and were not significantly higher than controls via Tukey HSD. This is probably due to low antibody titers at the 6.0 log_10_ TCID_50_/mL level. These results indicate that the Triton® X-100 treatment created significant differences in both ELISAs and the FMIA assay results, but did not affect FFN results.

## Discussion

The severity of disease caused by an outbreak of PEDV makes it imperative that an efficacious vaccine be developed. Due to the difficulties of *in vitro* cultivation and high virus transmissibility leading to biosecurity concerns, limited research has been performed in pigs in the U.S. With a four percent success rate for virus isolation being reported, the development of diagnostic tests and research of U.S. field strains has been hampered [6]. After successfully isolating and passaging a U.S. PEDV isolate, growth was maintained on M145 cells between 5.0-6.6 TCID_50_/mL.

The genetic characterization of NPL PEDV2013 P10.1 found that it is 99% identical to the strains circulating in Asia in the early 2010s. Its high genetic homology to the other circulating strains in the U.S. makes it a suitable candidate for investigation of U.S. PEDV inactivated vaccine immunogenicity in pigs. While there is data published regarding the efficacy of attenuated MLVs in Asia, there is limited published data on the immunogenicity of inactivated or subunit PEDV vaccines. This study demonstrates that inactivated PEDV vaccines are immunogenic is pigs.

Vaccine groups in this study were designed investigate at the effects of virus titer, site of administration and detergent treatment of antigen on immunogenicity in pigs. A dose response was observed by FFN for vaccines containing different virus titers, with 8.0 log_10_ TCID_50_/mL groups all being significantly greater than 6.0 log_10_ TCID_50_/mL groups. Vaccines were administered IM or in the rear flank to determine if the site of administration would affect overall immunogenic response. The flank vaccination site was only tested on the 8.0 log_10_ TCID_50_/mL groups, as we expected them to have the highest immune response; however there was no significant difference between the two sites of administration. Likewise, there was no significant difference between vaccines formulated with Triton® X-100 treated antigen, by FFN. A challenge model is needed to correlate FFN and/or ELISA titers to protection.

Surprisingly, with the exception of one pig, negative ELISA results were obtained from pigs vaccinated with Triton® X-100 treated virus formulated at 8.0 log_10_ TCID_50_/mL despite high FFN mean titers. The t-test shows Triton® X-100 treated groups were significantly different from non- Triton® X-100 treated groups at the same titer for ELISA and FMIA. Triton® X-100 detergent is used to create split-virion vaccines of influenza virus that are immunogenic and non-reactogenic [31]. Our data suggests that Triton® X-100 treatment of the PEDV antigen altered the antigenicity or immunogenicity of the NP, leading to negative ELISA results, while other immunogens capable of inducing a neutralizing antibody response detected by the FFN assay remained intact. The two ELISA tests and the FMIA all utilize NP antigen. Triton® X-100 treatment of the vaccine may have altered the conformation of the virion NP such that antibody induced by this antigen was not able to recognize the recombinant NP used in the ELISA or FMIA. An assay, such as the FFN, that detects functional neutralizing antibody associated with epitopes on the S protein may be better at quantifying antibody response to Triton® X-100 treated viruses. Few ELISAs targeting the S protein are readily available and they may also be subject to variations in protein conformation associated with Triton® X-100 treatment.

Though the vaccine in this trial was able to generate an antibody response, as indicated by FFN, FMIA, and ELISA assays, a protective titer is unknown. Previous work with attenuated virus used to vaccinate sows showed an immune response by ELISA in serum and colostrum, but could not draw a specific correlation to the level of mucosal immunity needed to confer protection [32]. Another study showed antibody was detected in serum from piglets and colostrum from pregnant sows after being inoculated with attenuated PEDV, though finding a specific protective antibody titer of the colostrum was complicated due to varying factors including litter size, colostrum uptake per piglet, antibody concentration, and quality of colostrum [20]. Due to biosecurity concerns, a post-vaccination challenge was not performed for this study. This should be done in the future to determine immune correlates of protection.

With reports that farms can be re-infected after a primary outbreak, disease will continue to be an ongoing problem due to lack of complete immunity after infection. In one case, piglets born from re-infected sows were reported to suffer around 30% mortality instead of near 100% during the first outbreak [33]. Further research is necessary to determine if secondary outbreaks in sows could be prevented via vaccination and or boosters. While this study focused on the humoral immune response in sera from vaccinated pigs, the post-vaccination immune response in sows and antibody titers in colostrum should be studied as the optimal vaccination regimen would utilize maternal antibodies to protect pigs when they are most susceptible to PEDV. Additionally, inactivated vaccines may prove efficacious when used as a booster in conjunction with live exposure or following MLV. If there is risk of re-infection among previously exposed herds, an inactivated vaccine booster to pregnant sows could reduce the occurrence of re-infection and limit secondary outbreaks. PEDV continues to be a source of economic loss and will continue to have a profound impact on the swine market in the U.S.

## Conclusions

These results demonstrate the immunogenicity of the PEDV inactivated viral vaccines with a U.S. strain. Information from this immunogenicity study shows the potential for inactivated vaccine development for U.S. PEDV strains. Further work is needed to evaluate protective FFN titers and ELISA and FMIA responses in a vaccination-challenge study.

## Abbreviations

PEDV: Porcine epidemic diarrhea virus
TCID50: Tissue culture infective dose
FFN: Fluorescent foci neutralization assay
FMIA: Fluorescent microsphere immunoassay
ELISA: Enzyme-linked immunosorbent assay
MLV: Modified live vaccine
S: Spike
ORF: Open reading frame
RFLP: Restriction fragment length polymorphism
USDA: United States Department of Agriculture
M145: MARC-145 cells
DMEM: Dulbecco’s modification of Eagles Medium
TPCK: L-1-Tosylamide-2-phenylethyl chloromethyl ketone
rt-RT-PCR: Real time reverse transcription polymerase chain reaction
IFA: Indirect immunofluorescent assay
SDSU: South Dakota State University
FITC: Fluorescein isothiocyanate
CPE: Cytopathic effect
IACUC: Institutional Animal Care and Use Committee
BEI: Binary ethyleneimine
IM: Intramuscularly
NVSL: National Veterinary Services Laboratory
MEM: Modified Eagles Medium
FFU: Fluorescent focus units
NP: Nucleoprotein
UMN: University of Minnesota
S/P: Sample to positive ratio
MFI: Mean fluorescence intensity
E: Envelope
M: Membrane
